# Supplementary material for: Effect of Benzotriazole on the Localized Corrosion of Copper Covered with Carbonaceous Residue
Source: Materials (Basel). 2021 May 21;14(11):2722. doi: 10.3390/ma14112722 (PMC8196688; doi:10.3390/ma14112722)
Supplement: Supplementary file 1 [file materials-14-02722-s001.zip › materials-1221573-supplementary.pdf]

materials

MDPI

Supplementary Information

Effect of Benzotriazole on the Localized Corrosion of Copper Covered with Carbonaceous Residue

Yun-Ho Lee [1], Min-Sung Hong [1], Sang-Jin Ko [1], and Jung-Gu Kim [1,*]

[1] School of Advanced Materials Science and Engineering, Sungkyunkwan University (SKKU), Suwon 16419, Korea

* Correspondence: kimjg@skku.edu (J.G.K); Tel.:+82-31-290-7360

2. Materials and Methods

2.2. X-ray Photoelectron Spectroscopy (XPS)

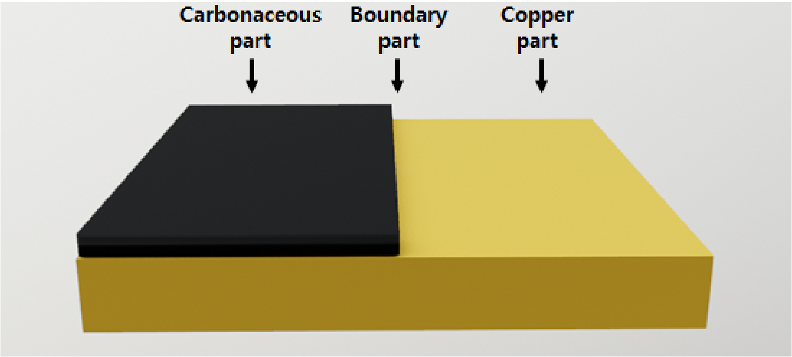

Figure S1. Schematic images of three parts (carbonaceous part, copper-carbonaceous film boundary part, and copper part) of specimen 2

3. Results and Discussion

3.1. Carbonaceous Film Analysis

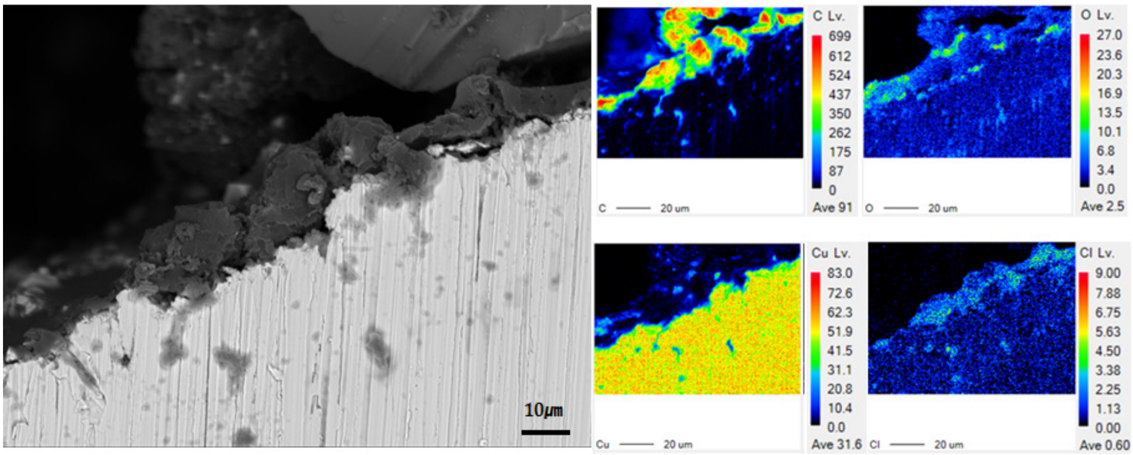

(**a**) (**b**)

Figure S2. Cross-sectional EPMA image near pitting corrosion of copper pipe for sprinkler used for 11 years; (a) EPMA image (1000x), (b) EPMA mapping

3.2. XPS Analysis

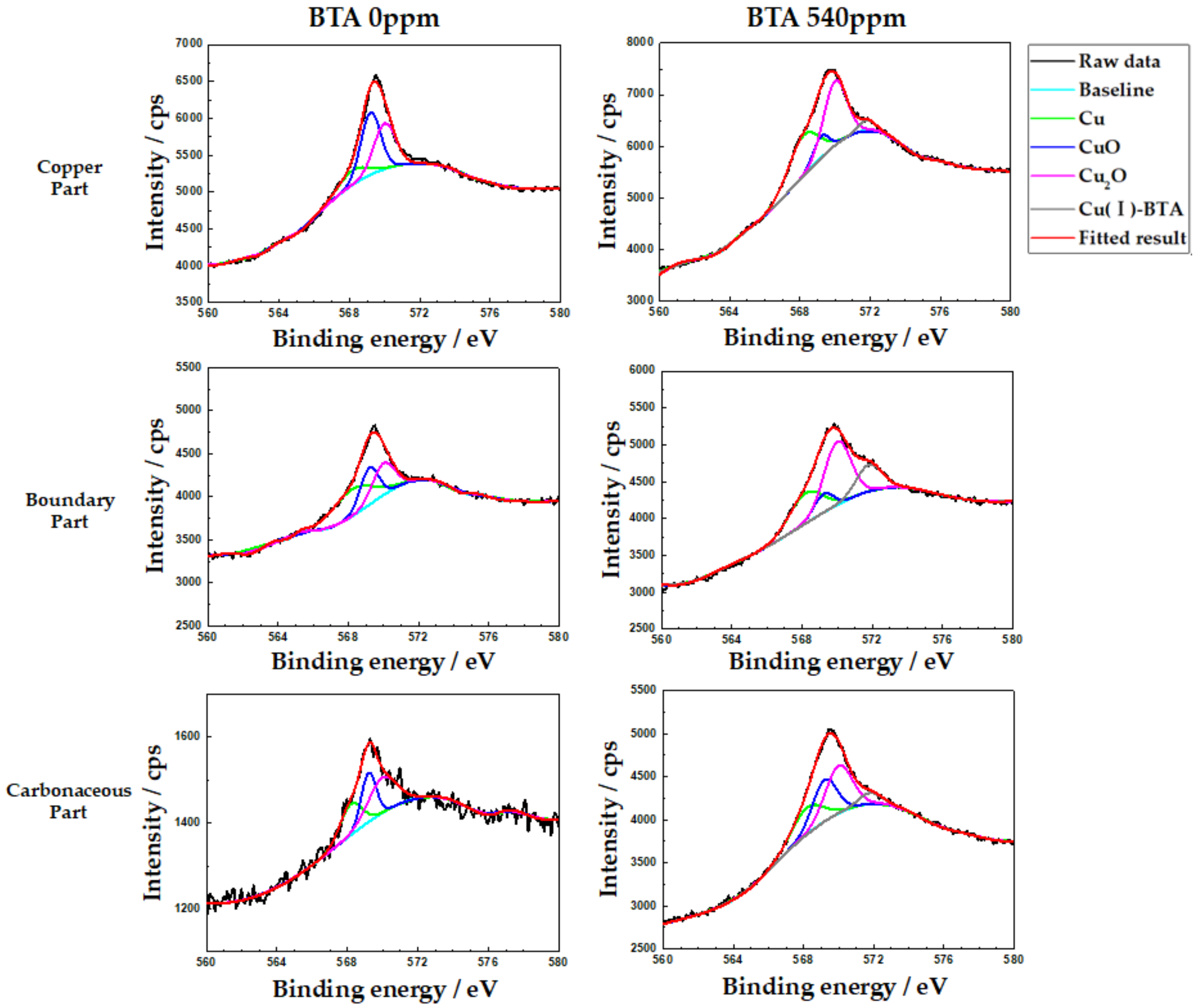

Figure S3. Cu LMM X-ray induced spectra and deconvoluted results at the surface of copper after 24-hour immersion in synthetic tap water at 25 °C based on the presence and absence of BTA.
